# Distinctive Cellular and Metabolic Reprogramming in Porcine Lung Mononuclear Phagocytes Infected With Type 1 PRRSV Strains

**DOI:** 10.3389/fimmu.2020.588411

**Published:** 2020-12-07

**Authors:** Elisa Crisci, Marco Moroldo, Thien-Phong Vu Manh, Ammara Mohammad, Laurent Jourdren, Celine Urien, Edwige Bouguyon, Elise Bordet, Claudia Bevilacqua, Mickael Bourge, Jérémy Pezant, Alexis Pléau, Olivier Boulesteix, Isabelle Schwartz, Nicolas Bertho, Elisabetta Giuffra

**Affiliations:** ^1^Université Paris Saclay, INRAE, AgroParisTech, GABI, Jouy-en-Josas, France; ^2^Aix Marseille Univ, CNRS, INSERM, CIML, Marseille, France; ^3^Genomics Core Facility, Institut de Biologie de l’ENS (IBENS), Département de biologie, École normale supérieure, CNRS, INSERM, Université PSL, Paris, France; ^4^Virologie et Immunologie Moléculaire, INRAE, Université Paris-Saclay, Jouy-en-Josas, France; ^5^Institute for Integrative Biology of the Cell (I2BC), CEA, CNRS, Université Paris-Sud, Université Paris-Saclay, Gif-sur-Yvette, France; ^6^Plate-Forme d’Infectiologie Expérimentale-PFIE-UE1277, Centre Val de Loire, INRAE, Nouzilly, France

**Keywords:** PRRSV-1, pig, lung mononuclear phagocytes, gene expression, immunogenomics, machine learning

## Abstract

Porcine reproductive and respiratory syndrome (PRRS) has an extensive impact on pig production. The causative virus (PRRSV) is divided into two species, PRRSV-1 (European origin) and PRRSV-2 (North American origin). Within PRRSV-1, PRRSV-1.3 strains, such as Lena, are more pathogenic than PRRSV-1.1 strains, such as Flanders 13 (FL13). To date, the molecular interactions of PRRSV with primary lung mononuclear phagocyte (MNP) subtypes, including conventional dendritic cells types 1 (cDC1) and 2 (cDC2), monocyte-derived DCs (moDC), and pulmonary intravascular macrophages (PIM), have not been thoroughly investigated. Here, we analyze the transcriptome profiles of *in vivo* FL13-infected parenchymal MNP subpopulations and of *in vitro* FL13- and Lena-infected parenchymal MNP. The cell-specific expression profiles of *in vivo* sorted cells correlated with their murine counterparts (AM, cDC1, cDC2, moDC) with the exception of PIM. Both *in vivo* and *in vitro*, FL13 infection altered the expression of a low number of host genes, and *in vitro* infection with Lena confirmed the higher ability of this strain to modulate host response. Machine learning (ML) and gene set enrichment analysis (GSEA) unraveled additional relevant genes and pathways modulated by FL13 infection that were not identified by conventional analyses. GSEA increased the cellular pathways enriched in the FL13 data set, but ML allowed a more complete comprehension of functional profiles during FL13 *in vitro* infection. Data indicates that cellular reprogramming differs upon Lena and FL13 infection and that the latter might keep antiviral and inflammatory macrophage/DC functions silent. Although the slow replication kinetics of FL13 likely contribute to differences in cellular gene expression, the data suggest distinct mechanisms of interaction of the two viruses with the innate immune system during early infection.

## Introduction

Although there have been several improvements in swine breeding, management, and health achieved in recent decades, porcine reproductive and respiratory syndrome (PRRS) remains one of the top challenges for the pig industry.

The causative virus (PRRSV) presents a vast genetic diversity worldwide and has been divided into two species, PRRSV-1 (European origin) and PRRSV-2 (North American origin) (1). The European subspecies PRRSV-1 is further divided into 4 genetic subtypes (2), and viral strains within these subtypes show strong differences in levels of virulence (3, 4).

In the lung tract, PRRSV mainly affects lung mononuclear phagocytes (MNP), in particular, alveolar macrophages (AM) and pulmonary intravascular macrophages (PIM) (5). Several immunoprofiling studies have been published on the effect of PRRSV infection on AM with comprehensive reviews provided (6, 7). Overall, cytokine expression and production are strain-dependent and differ between *in vitro* and *in vivo* conditions. *In vitro* differentiated macrophages and dendritic cells (DC) present variable susceptibility to PRRSV according to the culture conditions (8). *In vivo* primary DC subtypes do not support a productive replication, but the infection modulates their cytokine production and capacity to polarize T helper cells (9).

Early transcriptomic studies tried to highlight the complex and contrasting patterns triggered by different PRRSV strains (10–12). However, the heterogeneity of experimental designs, strains, and employed technologies have made it difficult to identify common and specific biological functions altered by PRRSV with only partial insights provided by the meta-analysis of available infection studies (13). Moreover, obtaining adequate transcriptional information for deriving biological interpretation can be challenging when dealing with low input amounts (e.g., specific cell subsets or single cells) as well as with low-virulent strains and early infection phases. AM is the most studied population with few other studies on peripheral blood mononuclear cells (14, 15) and tracheobronchial lymph nodes (16), but no genome-wide transcriptional studies have been reported yet for lung primary parenchymal MNP.

Classical statistics may be limited in handling data sets from biological studies using “Omics” methods (17). As a complement or alternative to classical approaches, supervised machine learning (ML) offers the possibility to accommodate heterogeneity of data and is robust to biological variation. ML helps to classify transcriptomic data by pattern recognition (expression “fingerprinting”) and provides mechanistic insights when paired with other downstream procedures, such as pathway analysis (18, 19). ML has been used in transcriptomic studies of viral infections in humans (20, 21) and was recently applied to identify genes associated with feed efficiency in pigs (22).

Here, we adopted an integrated gene expression approach to characterize different MNP subpopulations (macrophages and DCs) infected with the low-virulent Flanders 13 (FL13) strain (subtype 1.1) both *in vivo* and *in vitro* and analyzed a complementary *in vitro* experimental infection with high-virulent Lena (subtype 1.3). A low number of differentially expressed genes (DEGs) were found upon FL13 infection compared to mock-infected cells both *in vivo* and *in vitro* although the considerably higher number of DEGs triggered by the Lena *in vitro* infection confirmed the strong modulation induced by this strain. Classical DEG-based and gene set enrichment analysis (GSEA) approaches showed limited resolution on the *in vitro* FL13 data set, whereas ML provided additional biological insight into the gene expression modulation triggered by this strain.

Overall, our results indicate that the cellular and metabolic reprogramming differ between high and low pathogenic strains: FL13 keeps antiviral and inflammatory macrophage/DC functions silent, suggesting a different mechanism of pathogenesis upon early infection compared to the highly pathogenic Lena. This underlines the added value in biological interpretation of ML techniques for unraveling genomic signatures hidden with classical statistics approaches.

## Materials and Methods

### Virus Production and Titration

The two strains of PRRSV-1 used in this study, FL13 (13V091) (23) and Lena (3), were kindly provided by Dr. Hans Nauwynck (University of Ghent, Belgium). FL13 (13V091) was isolated in Belgium in 2013. This virus belongs to the usually low pathogenic pan-European subtype 1.1 but has spread rapidly in pig populations with good replicating capacity in macrophages (23). In subsequent *in vivo* and *in vitro* investigations, it did not reveal abnormal pathogenicity, and in our system, it behaved as a low pathogenic strain (personal communication) (9, 24). The Lena strain is a highly virulent strain belonging to the 1.3 subtype (2) isolated in Belarus in 2007 from a herd showing high mortality, reproductive failures, and respiratory disorders (3). Lena and FL13 viral stocks were produced using fresh AMs. Supernatants from infected cells were clariﬁed by centrifugation at 3,300 G, ﬁltered on 0.8 µm. Then, 30 ml of supernatant was layered on 4 ml 17% sucrose cushion and centrifuged at 100,000 G for 5 h, 30 min. The pelleted viruses were resuspended in RPMI medium. Titration of viruses was performed on fresh primary AM using the Spearman-Karber TCID50 method according to the OIE manual of diagnostic tests (OIE, “Chapter 2.8.7 Porcine Reproductive and Respiratory Syndrome,” Terr. Man., no. May 2015, 2015).

### *In Vivo* Infection

For the *in vivo* experiment, PRRSV-1 FL13 infection was performed at PFIE (INRAE, Nouzilly, France). The animal experiment was authorized by the French Ministry for Research (authorization no. 2015051418327338), and protocols were approved by the national ethics committee (APAFIS#413). Six large white female pigs coming from an INRAE breeding unit (Unité Expérimentale de Physiologie Animale de l’Orfrasière PAO-INRA, Nouzilly), free of respiratory infections and tested free from PRRSV, were housed in biosecurity level 2 air-ﬁltered animal facilities. Treatments, housing, and husbandry conditions conformed to the European Union Guidelines (directive 2010/63/EU on the protection of animals used for scientiﬁc purposes). At 8–9 weeks of age, three pigs were inoculated intranasally with FL13 (5x10^5^ TCID50/per animal in 2.5 ml per nostril), and three were mock infected. Five days postinfection (pi), the animals were euthanized (Zoletil, Virbac, France) and exsanguinated. Lung cells were collected and processed as previously described (9, 25, 26). Briefly, broncho-alveolar lavage (BAL) cells were collected using PBS + 2 mM EDTA. After the BAL procedure, peripheral parenchymal tissue from diaphragmatic lobes (PAR) were sampled, minced, and incubated in complete RPMI plus 2 mg/ml collagenase D (Roche, Meylan, France), 1 mg/ml dispase (Invitrogen), and 0.1 mg/ml Dnase I (Roche). Digested PAR was crushed and filtered on 100-µm cell strainers. Red blood cells were lysed using erythrolysis buffer (10 mM NaHCO_3_, 155 mM NH_4_Cl, and 10 mM EDTA). Cells were washed in PBS/EDTA and frozen in FBS + 10% dimethyl sulfoxide (DMSO, Sigma-Aldrich, St Louis, MO) for further analysis.

### Cell Sorting and Flow Cytometry Analysis

Isolated frozen BAL and PAR cells were separately enriched in MNP cells by 1.065 density iodixanol gradient (Optiprep^®^, Nycomed Pharma, Oslo, Norway) as previously described (9). They were stained in a blocking solution composed of PBS/EDTA supplemented with 5% horse serum and 5% swine serum. Antibodies were added to the blocking solution for 30 min on ice and then washed in PBS/EDTA with 2% FBS (for list of antibodies refer to **Supplementary Table 1**). Staining was accomplished in 4 steps: the uncoupled primary antibodies of different species/isotypes (mouse IgG1 anti CD13, mouse IgG2b anti-CD172a, mouse IgG2a anti MHC-II) followed by the secondary species/isotype speciﬁc ﬂuorochrome-coupled antibodies (anti-mouse IgG1-Alexa647, anti-mouse IgG2a-PE-Cy7, anti-mouse IgG2b APC-Cy7); then, the biotinylated primary antibody anti-CD11c followed by the streptavidin-coupled Alexa700 along with the ﬂuorochrome-coupled primary antibodies anti-CD163-PE and anti-CD1-FITC. A saturation step with IgG1, IgG2a, and IgG2b irrelevant antibodies was performed between the second and the third step. DAPI staining (Sigma-Aldrich) was performed to exclude dead cells. Compensations were set according to monocolor staining. The MoFlo ASTRIOS sorter (Beckman-Coulter, Paris, France) was used to isolate speciﬁc cell subpopulations. FlowJo software (version 10.1.0, Tree Star, Ashland, OR) was used to analyze subpopulation prevalence and purity. Cells were analyzed using an LSR Fortessa cytometer and Diva software (Becton Dickinson, Franklin Lakes, New Jersey) during standardization.

### *In Vitro* Infection

For *in vitro* experiments, lungs were obtained from the same breeding unit as for the *in vivo* study (Unité Expérimentale de Physiologie Animale de l’Orfrasière PAO-INRA, Nouzilly, France). All tested samples were negative for PRRSV. Lung parenchymal immune cells were collected and processed as described in the *in vivo* section. Parenchymal cells were then enriched in MNP cells by 1.065 density iodixanol gradient (Optiprep^®^, Nycomed Pharma, Oslo, Norway) as previously described (9). The cell composition of this gradient-enriched MNP preparation has been analyzed in detail in (9). The MNP was infected with either FL13 or Lena. All infections were performed on freshly enriched MNP at 2.10^6^ cells/ml for 24 h in complete RPMI at MOI 0.5. In order to avoid MNP adherence, incubations at 37°C were performed in 2-ml tubes with drilled caps for air renewal, located in a Corning-Costar 50-ml polypropylene tube. A complementary uninfected negative control was included.

### Low Input RNA-Seq Library Construction and Sequencing

RNAseq was performed on two sets of samples: 1) cDC1, cDC2, moDC, PIM, and AM cells from the *in vivo* experiments and 2) gradient-enriched MNP from the *in vitro* experiments.

Total RNA from cells was extracted directly after sorting using the Arcturus PicoPure RNA Isolation kit (ThermoFisher Scientiﬁc, Waltham, US) according to the manufacturer’s instructions. The RNase-Free DNase Set (Qiagen, Europe) was used to remove contaminating DNA. RNA integrity was assessed using an RNA 6000 Pico kit on a Bioanalyzer 2100 (Agilent Technologies, Santa Clara, US). RNA sequencing library preparation and Illumina sequencing were performed at the IBENS genomic core facility (Ecole Normale Superieure, Paris, France). Briefly, 1–3 ng of total RNA were amplified and converted to cDNA using the SMART-Seq v4 Ultra Low Input RNA kit (Clontech, Takara Bio Europe, Kyoto, Japan). Afterward, 150 pg of amplified cDNA were used to prepare the libraries with the Nextera XT DNA kit (Illumina, San Diego, US). The first set of libraries was sequenced in 30-plex on 3 high-output flow cells, and the second set was sequenced in 12-plex on 1 high-output flow cell. A 75-bp read sequencing was performed on a NextSeq 500 sequencer (Illumina, San Diego, US). Respectively, a mean of 50 ± 5.7 and 40 ± 3.7 million passing Illumina quality filter reads was obtained for samples from the first and second experiments.

### RNAseq Data Analysis

The analyses were performed using the Eoulsan pipeline (27), which included the steps of read filtering, mapping, alignment filtering, read quantification, normalization, and differential analysis. Before the mapping step, poly *N* read tails were trimmed, reads shorter than 40 bp were removed, and reads with a mean quality ≤30 were discarded. Reads were then aligned against the *Sus scrofa* 11.1 genome from Ensembl version 91, FL13 (GenBank accession number: KT159248) or Lena virus (GenBank accession number: JF802085.1) using STAR (version 2.5.2b) (28). Alignments from reads matching more than once on the reference genome were removed using the Java version of samtools (29). To calculate gene expression, the *Sus scrofa* GTF genome annotation version 91 from the Ensembl database was used. All overlapping regions between alignments and referenced exons (or genes) were counted using HTSeq-count 0.5.3 (30).

The RNAseq gene expression data and the raw FASTQ files were submitted to GEO (www.ncbi.nlm.nih.gov/geo/) and are available under the accession number GSE147632.

The normalization step and the differential analyses were carried out with DESeq2 1.8.1 (31). The expression matrix thus obtained was then filtered to retain the genes with a minimum 2-fold change across all samples and used to perform a principal component analysis (PCA) with FactoMineR (32) and a clustering analysis subject to multiscale bootstrap resampling with Pvclust (33) in the R statistical environment.

### Machine Learning

To perform ML of the FL13-infected and control MNP *in vitro* data sets, the normalized count matrix and the attributes (genes) obtained from each replicate (*n* = 4) were used. The normalized count matrix was used for both classical statistics and ML approaches. A first data reduction was performed by removing genes without expression and genes with no consistent replicate values, which generated approximately 16,000 genes that were subsequently used for the downstream ML approach.

The Waikato Environment for Knowledge Analysis (WEKA version 3.8.4, www.cs.waikato.ac.nz/ml/weka/) was used to perform the sequential minimal optimization (SMO) support vector machines (SVMs) learning classifier algorithm (34) together with decision tree approaches (random forest or RF and J48 algorithms) and multilayer perceptron (MLP) neural network (19). Genes were ranked based on Shannon entropy (information gain) in dichotomous classification assignment by SVMs (35). The SVMs, RF, and J48 were used to classify the FL13- and mock-infected gene data set in a data dimensionality reduction approach as previously described (36). MLP was used when the data set was reduced to below 2,000 genes due to the high computing capacity needed for the algorithm.

Class assignment of all ML algorithms was evaluated by two cross-validation strategies: 1) a percentage split whereby 66% of the data were randomly selected and used for training and the remaining 34% of the data were used in testing and 2) a stratified hold-out (*n*-fold) method with 4-fold, in which 3-fold of the randomized gene expression data were used for training and 1-fold was used for testing. A 4-fold stratified holdout was used for SVMs, RF, and J48 cross-validation when the samples were classified as FL13 or control using the complete gene expression data set. In all cross-validations, the SVMs, RF, and J48 were naive to the holdout data and the percentage of correct assignment was used to evaluate classifier performance. All data classes were properly represented in the ML training and cross-validation data sets (37). The performance of the SVMs, RF, and J48 was evaluated as a percentage of correct classification during the cross-validations. Additionally, the Kappa statistic and area under the receiver operating characteristic curve (AUROC) of each model were reported. Kappa statistic and AUROC evaluate the agreement between the actual and assigned classes by a classifier and can be interpreted in a way similar to a *p*-/*q*-value of classical statistics (19). The random probability of chance for dichotomous assignment was assumed to be 50% based on the law of probability. A negative Kappa statistic and/or an AUROC of less than 0.500 indicates that ML classifier performance is worse than that predicted by random assignment. As a negative control of ML, the values for the 900 relevant gene data sets were randomly reordered 10 times, and each was entered into ML and analyzed with SVMs, RF, and J48 as described above. The results of these negative control models were interpreted based on expectations of the law of probability.

### Generation of Cell-Specific Gene Signatures and High Throughput GSEA

All the murine microarray data used in the study were obtained from GEO and generated using the Affymetrix Mouse Gene 1.0 ST array (Thermo Fisher Scientific, Waltham, US). Three data sets were used to match as much as possible the diversity of the cell types studied. We selected the data sets in such a way that at least one cell type was found in common in order to enable validation of data set effect correction. Two data sets were generated by the Immgen consortium (GEO accession numbers GSE15907 and GSE37448), and a third data set was generated by the Murphy laboratory [GSE75015, (38)].

The list of microarray samples used, their GEO identification numbers, and expected associated cell populations are provided in **Supplementary File 1** (murine_microarrays_EC_study.csv).

Raw CEL files were processed in the R statistical environment (version 3.6.1). Quality control of the array hybridization and normalization of the raw expression data with robust multi-chip analysis (39) were performed using the “oligo” R package (40). Presence of a putative batch effect was evaluated by a PCA, which revealed an effect due to data sets. This was corrected using the ComBat batch correction algorithm from the “sva” R package (41).

BubbleGUM software (42) was used to generate splenic and lung MNP subset and porcine blood (GSE66311) cell subset specific transcriptomic signatures and to assess their enrichment across pig lung cell types in a similar way as in Carpentier et al. (43).

Using GeneSign, gene signatures of murine and porcine cell subsets were generated (i.e., the lists of genes that are more highly expressed in the cell subsets of interest as compared to other cell subsets) using the “MinTest/MaxRef” calculation method with a minimal fold change of 1.0 or 1.2 (home_made_signatures_PIG_symbols_EC_study.csv, **Supplementary File 2**) (porcine_blood_cell_signatures_EC_study.cvs, **Supplementary File 3**). The murine MNP signature Affymetrix IDs were then converted into the official symbols of their porcine gene orthologs using Ensembl BioMart (44) (**Supplementary File 2**).

To run BubbleMap, we used these signatures together with a list of 50 independent GeneSets from MSigDB (45) for statistical power. BubbleMap was used with 1,000 gene set–based permutations and with “signal to noise” as a metric for ranking the genes. The results are displayed as a BubbleMap, in which each bubble is a GSEA result and summarizes the information from the corresponding enrichment plot.

### Unsupervised Hierarchical Clustering and Multiscale Bootstrap Resampling

For the transcriptomic data, only the genes displaying a fold change >2 across all conditions were considered. The robustness of the tree was tested by multiscale bootstrap resampling using the pvclust package with parameters “correlation” as distance and “Ward.D2” as cluster method with 1,000 iterations at 10 different data set sizes comprising between 50% and 140% of the complete data set.

### Functional Analyses of the Transcriptome

Functional analyses of swine RNAseq data were performed using two approaches. First, all the DEG lists obtained from both *in vivo* and *in vitro* experiments and the gene list obtained by ML were analyzed with Ingenuity Pathways Analysis 01-16 (IPA, version 51963813, March 2020 release, Qiagen, Hilden, Germany). Only the canonical pathways with a –log (*p*-value) >2 and including at least 4 genes were considered. A second analysis was performed using GSEA (version 4.0.3, http://software.broadinstitute.org/gsea/index.jsp). The analysis was carried out using the “h.all.v7.1” and “c2.cp.kegg.v7.1” MsigDB gene sets, with 2,500 permutations and the “gene_set” option as permutation type. Prior to both analyses, the porcine gene names were converted into their human orthologs using the “dbOrtho” tool of bioDBnet (https://biodbnet-abcc.ncifcrf.gov/db/dbOrtho.php) in order to match the MSigDB gene set human symbols.

### RT-qPCR

Quantification of viral particles using RT-qPCR detection was performed in swabs and sera, using FL13 N gene primers (F: 5’ GGGAATGGCCAGTCAGTCAA 3’, R: 5’ ATCTTCAGCAGCTAGGGGGA 3’) (46) and setting a limit of detection at Ct = 35. Briefly, viral RNA was extracted using the NucleoSpin^®^ RNA Virus kit (Nagel-Macherey GmbH, Düren, Germany). A one-step qRT-PCR was performed using the iTaqTM universal probes one-step kit (BIO-RAD, Hercules, CA, USA). A standard curve was made with four 10-fold dilutions of the FL13 inoculum (10^1^ to 10^4^ TCID50/mL). The number of TCID50-equivalent/mL in each sample was determined using the FL13 RNA standard calibration curve.

Based on the transcriptomic analyses, a panel of porcine genes was selected for qPCR validation of RNAseq data. Primers were designed with PrimerExpress 3.0 using standard parameters (Thermo Scientific, Waltham, US), and the potential formation of primer dimers was further evaluated using the “Multiple Primer Analyzer” web tool (Thermo Scientific, Waltham, US). Primer sequences are provided in **Supplementary Table 2**.

The qPCR validations were performed on residual cDNA after RNA-seq library preparation. The cDNA concentrations were estimated with a Qubit (Thermo Fisher Scientific, Waltham, US) and normalized to 80 pg/µl in a final volume of 100 µl.

Amplification reactions were run in duplicate on a QuantStudio™ 12K Flex (Thermo Fisher Scientific, Waltham, US). First, we validated primer efficiency with a standard curve of four serial dilution points of cDNA (ranging from 1,000 pg to 1 pg of cDNA) and a no template control (NTC). The qPCR amplification mixture (20 µL) contained 5 µL single-strand cDNA template (400 pg for each reaction), 10 µL 2X Power SYBR Green PCR Master Mix buffer (Thermo Fisher Scientific, Waltham, US), and forward and reverse primer (final concentration of 100 nM each) for the genes *BIRC5, ATP2C1, COX5B, ATG5, ENS13436 (CYT B-C1-10), CYC1*, and *SHARPIN* and forward and reverse primer (final concentration of 300 nM each) for the genes *STMN1, DTX4, COX5A*, and *GAPDH*. After primer optimization (with range of efficiency -3.32 to -3.4), the qPCR reactions were run in triplicate on a QuantStudio™ 12K Flex (Thermo Fisher Scientific, Waltham, US), and the quality control of data was performed using the Connect platform (Thermo Fisher Scientific, Waltham, US). The data were subsequently downloaded as .txt files and analyzed by the ΔΔCt method.

The correlation among the fold change values obtained by qPCR and the corresponding ones obtained by RNAseq was evaluated using a Spearman’s Rho test.

## Results

### FL13 *In Vivo* Infection

Pigs were infected or mock-treated with FL13, and nasal swabs and sera were collected at days 0 and 5 pi. As expected, at 5 days pi, the swabs and sera of FL13-infected animals were positive (between 50 and 5,000 TCID50-equivalent/mL; **Supplementary Figure 1A**). Animals did not show evident clinical signs, and lungs presented only mild lesions at culling (data not shown), which was compatible with the early stage of the infection and with the low pathogenicity of the strain.

### Isolation and Sorting of Enriched MNP From Lung Tissues

**Figure 1:**
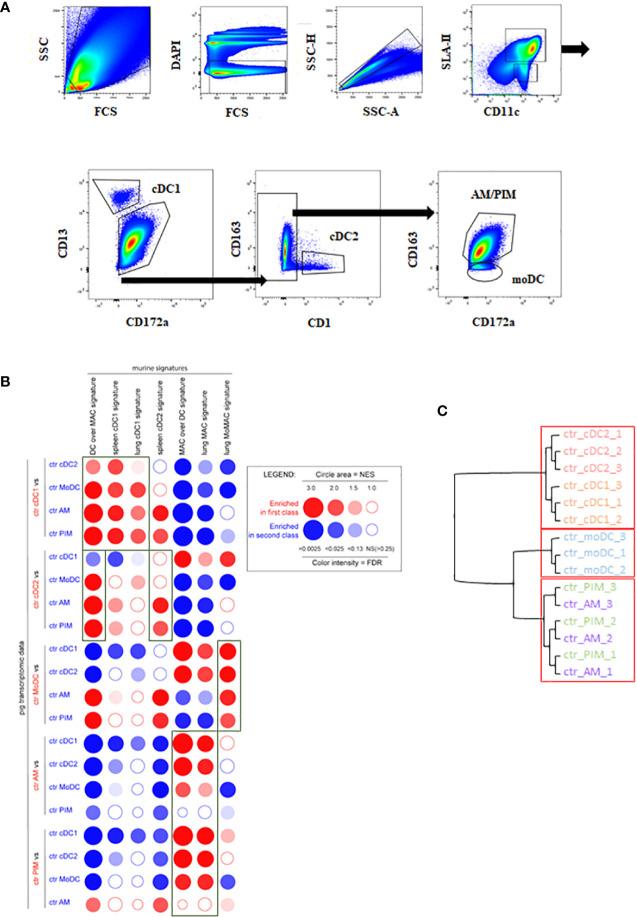
MNP subpopulations sorting strategy *in vivo*. cDC1, cDC2, moDC, AM, and PIM were sorted from infected or control lung parenchyma at day 5 pi. **(A)** MNP subpopulations were defined by using MHC-II/CD11c/CD172a/CD13/CD163 markers. Each graph is the offspring of the previous gate from left to right or following the arrows. Data are representative of 3 independent sorting experiments. **(B)** Analysis of the homologies between porcine and mouse MNP subsets by high throughput GSEA using BubbleGUM. Data are represented as bubbles, bigger and darker for stronger and more significant enrichment in a color matching that of the population in which the gene set was enriched. Boxes correspond to enrichments of interest. **(C)** Unsupervised hierarchical clustering and multiscale bootstrap resampling on the transcriptome of sorted MNP from control animals. An approximately unbiased (AU) *p*-value was calculated for each node of the cluster dendrogram. Clusters with AU ≥ 95% are indicated by the rectangles.

Enriched MNP isolated from BAL or from lung parenchyma (100–200.10^6^/sample) were stained with a 7-color flow cytometry strategy as described in the Materials and Methods section, following the strategy used in (9), to sort AM, PIM, moDC, cDC1, and cDC2 (25). Because of a 1-year-long commercial anti-CADM1 antibody shortage in the United States and Europe, the protocol was modified to use CD13 instead of CADM1 to gate cDC1. We took advantage of the identification of CD13 as a cDC1 marker in bovine blood (47) and human lung (48) to use antiswine CD13 monoclonal antibodies generated in our laboratory (49). The new cDC1 gating was validated as MHCII+, CD11c+, CD13+, CD172a-, and CD163- (**Figure 1A**) and allowed the identification of the very same cells as the one stained using anti-CADM1 antibodies (**Supplementary Figure 2**).

After sorting, 10^5^ cells per subtype were obtained except for the cDC1 and cDC2 subsets for which the cell number ranged between 1,800 and 63,000 cells, depending on the sample.

### Transcriptional Signatures of MNP Subsets

A total of 30 cDNA libraries from the cell populations of the two groups (FL13-infected and control) were sequenced, which yielded 50 ± 5.7 M reads/sample mapped to the pig genome.

The RNAseq data of the different porcine pulmonary MNP were compared, using BubbleGUM with murine MNP signatures in order to validate the sorting strategy (42).

By performing pairwise comparisons of porcine immune cell types, we examined the distribution bias of transcriptomic signatures specific for putatively equivalent mouse immune cells using the GSEA methodology as previously published for the comparison of human and mouse cell types (50) (**Figure 1B**).

The mouse DC over MAC [i.e., genes more highly expressed in murine DC as compared to mouse lung macrophage (MAC)] signature was systematically and strongly enriched in DC subsets (cDC1, cDC2) and moDC when compared to macrophages (AM, PIM), thus validating the DC identity of these 3 cell types. Moreover, the DC over MAC signature was enriched in cDC1 and cDC2 when compared to moDC. The mouse spleen cDC2 signature was systematically enriched in porcine cDC2 although non significantly when compared to cDC1 and moDC, whereas spleen and lung cDC1 signatures were systematically enriched in cDC1 versus all the other lung MNP. The lung MAC signature was significantly enriched in porcine AM except for comparison with PIM. Similarly, the mouse MAC over DC signature (i.e., genes more highly expressed in murine macrophages as compared to DC) was significantly enriched in porcine AM except for comparison with PIM, in agreement with the absence of this subpopulation in mice (5, 51). The mouse monocyte-derived cell signature (lung MoMAC) was significantly enriched in moDC in agreement with a differentiation from blood monocytes of porcine moDC (**Figure 1B**). Alternatively, we also compared porcine lung MNP transcriptomic data with porcine blood mononuclear cell signatures generated from data obtained in a previous work (**Supplementary Figure 3**) (52). The cDC1 blood signature was clearly enriched in the lung cDC1 when compared to all other lung cell subsets. The classical monocyte (cMo) signature was enriched in the moDC, PIM, and AM when compared with cDC, confirming the monocyte–macrophage functional bias. The population defined as nonclassical monocytes (ncMo) resembled more the moDC population. Finally, the B lymphocyte signature was not enriched in our lung cells, and the pDC signature had very little enrichment in lung DC subsets when compared with AM and PIM.

Additionally, to test the similarity of expression patterns between MNP replicates, a hierarchical clustering was performed on the pulmonary MNP data (**Figure 1C**). As expected, the approach showed a clear segregation between lung tissue-resident macrophages (AM and PIM) and lung cDC (cDC1 and cDC2). The clustering put in evidence the clear difference between cDC and moDC because moDC preferentially clusters with AM and PIM, whereas AM and PIM did not cluster separately, highlighting their similarity and probable common origin (**Figure 1C**) (5). The AM versus PIM contrast showed the lowest number of DEGs, and all the other contrasts highlighted high differences between the diverse cell subtypes (**Supplementary Table 3A**).

Overall, the high-throughput GSEA using murine and blood porcine signatures validated and confirmed the identities of the porcine lung MNP isolated with the new sorting strategy.

### Mild Effects of FL13 Infection on MNP Subsets *In Vivo*

We investigated the level of infection in each cell subtype by mapping the sequences to the FL13 reference genome sequence. As expected, AM and PIM showed the highest level of FL13 matching reads compared with DC subtypes (**Supplementary Figure 1B**). The moDC, cDC1, and cDC2 cells showed low levels of FL13 matching reads (45–480 reads), which is compatible with either PRRSV abortive infection, endocytosis of viral particles, or phagocytosis of infected apoptotic cells by the three DC subtypes and comparable to our previous results (9).

To evaluate the impact of FL13 infection on the different MNP subsets, we performed PCA (**Supplementary Figure 4A**) and a hierarchical clustering (**Supplementary Figure 4B**) on the transcriptomes of MNP from control and infected animals. Both approaches showed a clear lack of separation between cells from FL13-infected and control animals, highlighting the scarce modifications induced upon FL13 infection that are probably related to the low pathogenicity of the strain and/or to the mild effects at the early stage of infection.

### Evidence of Cell Cycle Arrest and Apoptosis in FL13 Infected-AM and PIM *In Vivo*

**Figure 2:**
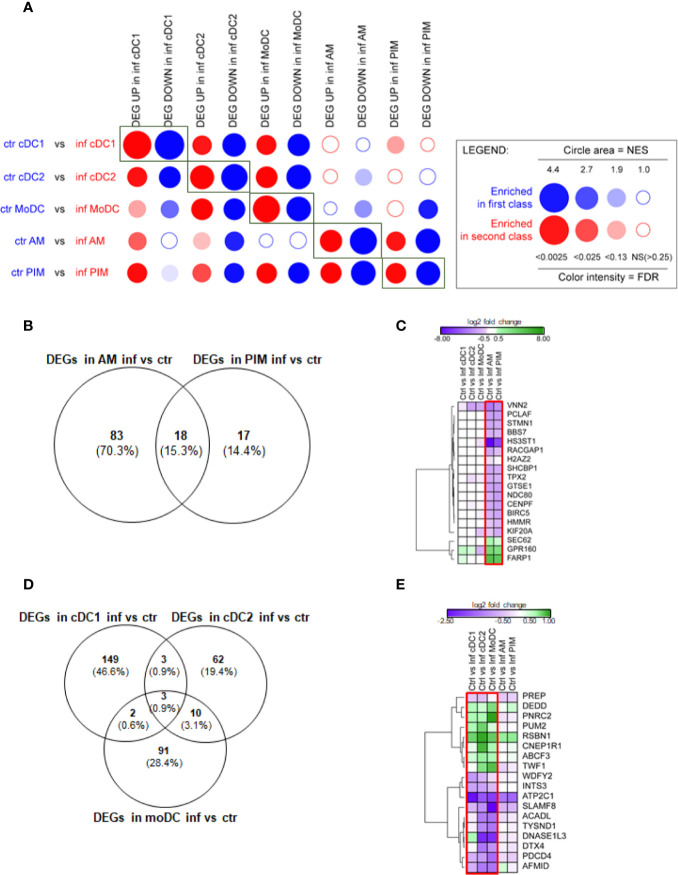
FL13 *in vivo* infection. Macrophages (AM, PIM) and DC subsets (moDC, cDC1, and cDC2) sorted from FL13 (inf) and control (ctr) animals at day 5 pi. **(A)** Analysis of the DEGs between the different MNP subsets by high throughput GSEA using BubbleGUM. Control/validation enrichments, in which genes identified as DEGs in a given cell subset between control and FL13 infected animals were found enriched by BubbleMap in the respective counterparts, are highlighted by rectangles. **(B)** Venn diagram of DEGs identified in all macrophage subsets based on the contrast of AM and PIM from infected versus control animals and a FDR < 0.05. **(C)** Heatmap displaying the log2 ratios of the DEG shared between AM and PIM from infected versus control animals as shown in **Figure 2B**. Three and 15 DEGs are up- (green) and down- (purple) regulated, respectively. Red box includes DEGs with FDR < 0.05. **(D)** Venn diagram of DEGs identified in all 3 DC subsets based on the contrast of cDC1, cDC2, and moDC in infected versus control animals and a FDR < 0.05. **(E)** Heatmap displaying the log2 ratios of the DEGs shared between at least two DC subsets from infected versus control animals as shown in **Figure 2D**. DEGs are up- (green) and down- (purple) regulated. Red box includes DEGs with FDR < 0.05 in at least two DC subtypes.

In order to gain insight into the similarities and differences each MNP subset transcriptionally undergoes upon FL13 infection, we performed a BubbleMap analysis, using as gene sets the DEGs identified in each MNP subset upon infection and comparing each MNP subset from control and infected animals (**Figure 2A**). This analysis revealed that the genes that were significantly up- and down-regulated in cDC1, cDC2, and moDC from infected animals were also found to be globally up- and down-regulated in all other MNP from infected animals, including AM and PIM, although to a lesser extent. However, genes found that significantly up- and down-regulated in AM and PIM were globally also found similarly regulated in PIM and AM, respectively, but not in DC subsets, most likely because these genes are modulated by the PRRSV productive infection of PIM and AM (9) (**Supplementary Figure 1B**). Interestingly, the genes up-regulated in moDC upon infection were not, as a group, found regulated in the AM subset (**Figure 2A**) in agreement with the respective pro- and anti-inflammatory functions of moDC and AM.

When focusing on DEGs (FDR < 0.05), AM from FL13-infected animals showed 101 DEGs compared to AM from control animals, whereas PIM showed only 35 DEGs in infected versus control animals (**Figure 2B**). Thus, PRRSV-1 triggered a limited gene modulation on PIM compared to AM although they presented similar virus levels [**Supplementary Figure 1** and (53)]. Interestingly, upon FL13 infection, PIM shared more than half of their DEGs (18, of which 15 down-regulated and 3 up-regulated) with AM (**Figure 2C**). Log2 FC levels of DEGs were similar between AM (Mean -1.22 ± 2.7 SD) and PIM (Mean -1.13 ± 1.8 SD).

*BIRC5* and *STMN1* were validated by qPCR (**Supplementary Table 4**). *BIRC5* [a member of the inhibitor of apoptosis protein (IAP) family that prevents apoptotic cell death] was down-regulated together with 7 other genes (*CENPF, GTSE1, HMMR, KIF20A, NDC80, STMN1*, and *TPX2*) involved in the progression of the cell cycle, mitosis, and DNA damage responses. Among them, *STMN1* was identified by meta-analysis of PRRV data (13). Thus, our data align with previous studies showing that PRRSV modulates apoptosis (54) and cell cycle in AM while integrating, for the first time, the analysis of the PIM expression.

### Upon FL13 Infection, DC Showed Cell Cycle Arrest and an Anti-Apoptotic Profile *In Vivo*

The number of DEGs found in cDC1 (157) from infected animals was higher than in moDC (106) and cDC2 (55) (**Figure 2D**) (**Supplementary Table 3B**).

Only three DEGs (*ATP2C1, INTS3, RSBN1*) were shared between the three DC subtypes (**Figure 2D**). *ATP2C1* (validated by qPCR, **Supplementary Table 4**) codes for the SPCA1 protein, an ATP-powered calcium pump that transports calcium ions into the Golgi. SPCA1 regulates proteases within the *trans-*Golgi network that require calcium for their activity and have been described as critical for the maturation of different virus glycoproteins (56). The tendency (*p* value > 0.05) of *ATP2C1* down-regulation found in macrophages suggests that it might be involved, but not sufficient, in the decreased virus susceptibility described in *in vivo* DC subtypes (9).

Upon infection, DC displayed the down-regulation of a gene related to the DNA repair and mitotic cell cycle, *INTS3*, and the up-regulation of *RSBN1*, a gene involved in chromatin organization.

Three DEGs were exclusively shared by cDC1 and cDC2 from FL13-infected animals (**Figure 2E**), all involved to a certain extent with protein and peptide cellular turnover: *PUM2*, a protein involved in the translational control, acting as a post-transcriptional repressor by binding mRNA targets; *PREP*, a cytosolic endopeptidase with proteolysis function and *ENSSSCG00000040538* (*WDFY2*), a protein and metal ion-binding gene.

MoDC shared several DEGs (11) with cDC2 but only 2 DEGs with cDC1 (**Figures 2D, E**). The two shared with cDC1, *DEDD* (a regulator of the apoptosis process, a scaffold protein that directs CASP3 to substrates and facilitates in their ordered degradation during apoptosis) and *PNRC2* (a mediator of mRNA decay) were up-regulated. Only 3 (*ABCF3, CNEP1R1, TWF1*) out of 11 genes shared between moDC and cDC2 were up-regulated in response to infection.

Although the expression profile of macrophages (AM and PIM) from infected animals suggested apoptosis, moDC and cDC2 profiles presented a general down-regulation of apoptosis promoters, such as *DNASE1L3* (which promotes DNA breakdown during apoptosis) and *PDCD4* (promoter of apoptosis and autophagy) upon infection. We also observed the down-regulation of *DTX4* (validated by qPCR, **Supplementary Table 4**), an important promoter of the innate immune system and regulator of the Notch signaling, involved in DC differentiation (57) and part of the mechanism of degradation of TBK1, belonging to the type I interferon signaling (58).

Finally, *ABCF3* has been classified in the defense response to virus GO term. Its silencing has been correlated with increased replication of flavivirus (59). *ABCF3* up-regulation mainly in cDC2 and moDC might represent another complementary molecular mechanism to control PRRSV replication in DC. Interestingly, two upregulated DEGs shared by all DC contrasts, i.e., PUM2 and TWF1, had been related to the response to different PRRSV strains by meta-analysis (13).

### Pathway Analysis of *In Vivo* FL13 Infected-Cell Subsets

**Figure 3:**
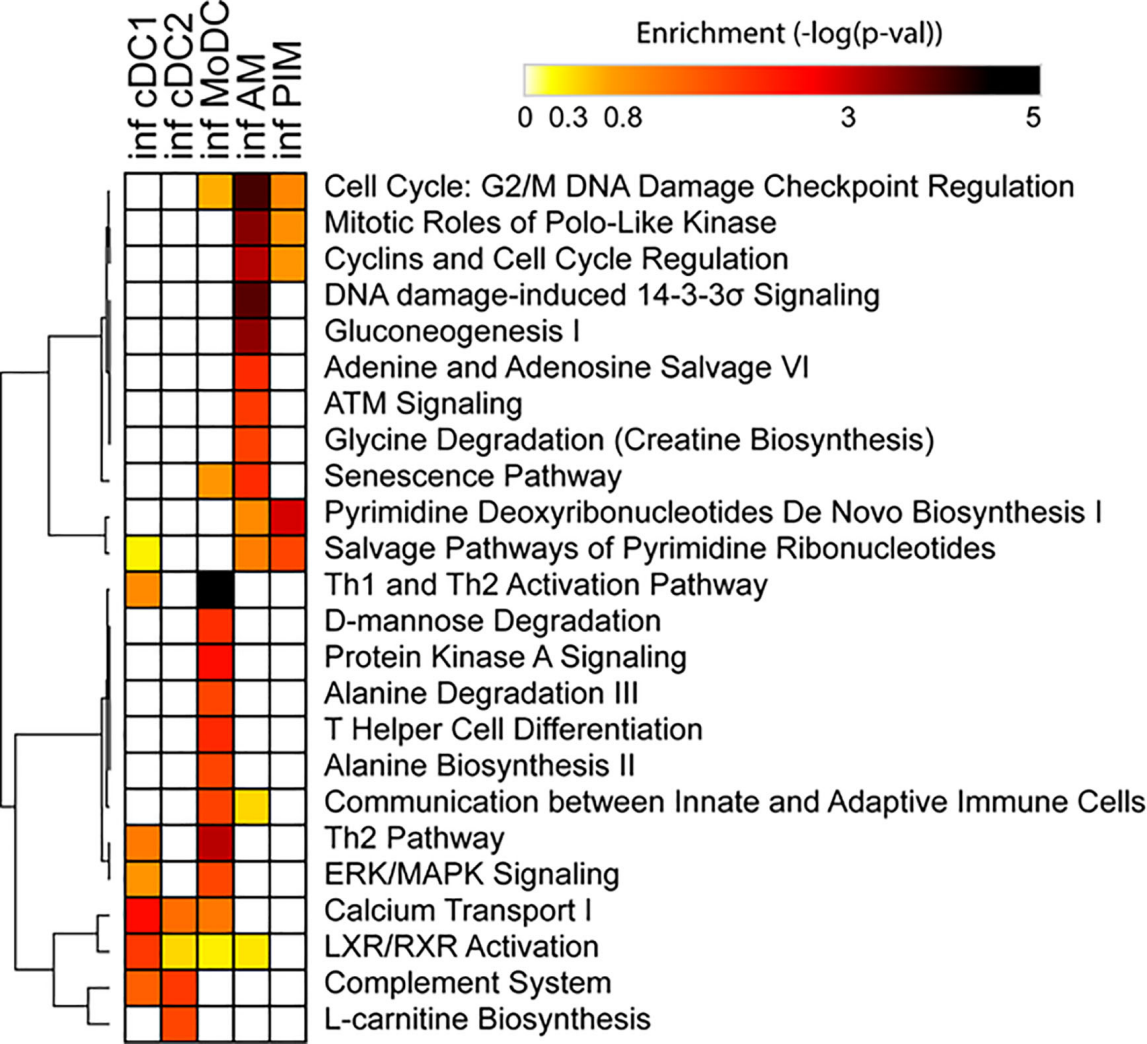
Enrichment of functional annotations in MNP subsets from infected versus control animals using IPA. Heatmap displaying selected functional annotations from IPA found to be enriched in one or more cell subsets from FL13-infected versus control animals. Hierarchical clustering was performed with Morpheus (Broad Institute) using the One minus Pearson correlation and the average linkage method.

**Figure 4:**
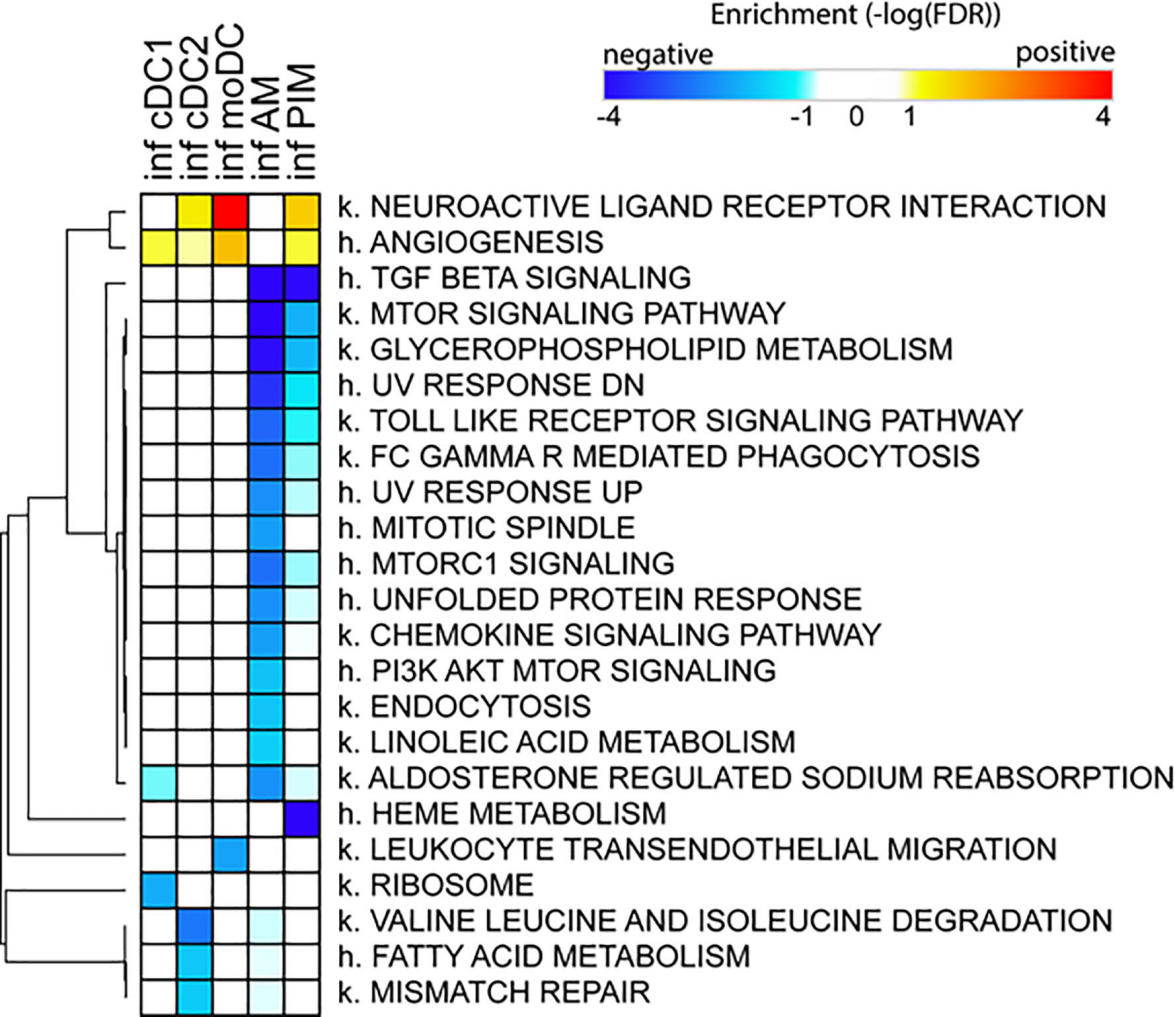
Enrichment of functional annotations in MNP subsets from infected versus control animal using GSEA. Heatmap displaying selected gene sets from MSigDB found to be enriched by GSEA in one or more cell subsets from FL13-infected versus control animals. Gene sets found up- and down-regulated upon infection have positive (red) and negative (blue) enrichments, respectively. Gene sets preceded with k. come from the Kegg pathway database, and gene sets preceded with h. come from the Hallmark collection of the MSigDB. Hierarchical clustering was performed with Morpheus (Broad Institute) using the One minus Pearson correlation and the average linkage method.

Due to the low number of DEGs, using IPA, we detected only a few significant pathways in the FL13 PIM versus control contrast that were mainly related to the cell cycle biological process. Similarly, the FL13 AM versus control contrast showed pathways involved in cell cycle process and senescence together with additional metabolic pathways (**Figure 3**). The GSEA approach (**Figure 4**) confirmed the IPA findings related to the down-regulation of cell cycle/mitosis/replication and DNA/nucleotide repair capacity in infected AM/PIM and allowed limited additional biological interpretations. AM from infected animals showed down-regulation of the mTOR pathway, unfolded protein response, and phagocytosis function together with the reduction of metabolic pathways related to their altered energetic capacity upon infection. These features were shared between AM and PIM with the data clearly showing that AM were the most affected subset (**Figure 4**). Thus, both IPA and GSEA pointed to altered cell cycle, DNA repair, and altered metabolism in cells from infected animals together with the apoptotic phenotype of AM and PIM upon FL13 infection.

When we consider the DC subsets, IPA detected only few significant pathways with moDC displayed as the most affected population upon infection (**Figure 3**). MoDC versus the control data set was enriched in several metabolic pathways and, interestingly, in the “Th2 pathway” and “Th1 and Th2 activation pathway,” which suggests a potential alteration in their T cell priming ability (**Figure 3**). In this case, GSEA was not able to increase any significant biological interpretation (**Figure 4**). Thus, both IPA and GSEA of DEGs allowed limited insights into the transcriptional regulation of DC subsets triggered by PRRSV-1.

### FL13 and Lena *In Vitro* Infection of MNP

We previously reported that *in vitro* infection of enriched parenchymal lung MNP preparations follows a similar infection pattern to *in vivo* infected MNP (9). Thus, fresh parenchymal lung cells were enriched in MNP by density gradient and infected with either FL13 or Lena virus. Lena infection was added into the system to gain comparative insights with a highly pathogenic strain using a refined primary *in vitro* system.

It is important to note that MNP prepared from lung parenchyma after the BAL procedure are depleted of most of the AM (26).

As for the *in vivo* study, we investigated the level of infection in each group by first mapping viral reads to the FL13 and Lena reference genomes. As expected, Lena showed the highest level of matching reads compared with FL13 (**Supplementary Figure 1C**).

Subsequently, the host transcriptome profiles were analyzed between FL13- and Lena-infected cells and controls (**Supplementary Table 3C**). Only 24 DEGs were found in FL13-infected cells compared with controls, whereas a huge amount of DEGs (4,556) was found after Lena infection (**Supplementary Table 3C**). This confirmed previous reports of the strong immune stimulation induced by Lena (9, 60, 61).

Similar to the *in vivo* setup, FL13 *in vitro* infection of MNP triggered limited immune cell stimulation.

**Figure 5:**
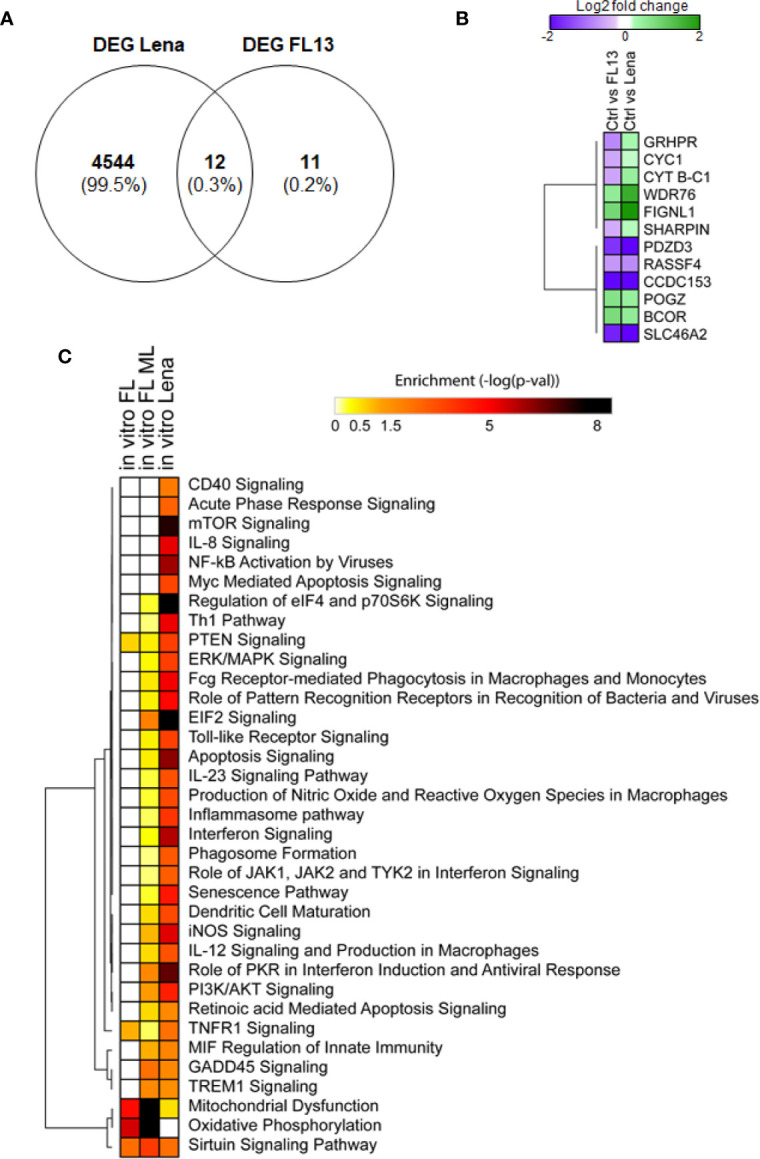
FL13 and Lena *in vitro* infection. Enriched MNP were infected with FL13 and Lena at MOI = 0.5 and mock inoculated for 24 h. **(A)** Venn diagram of DEGs between FL13 and Lena infected versus control MNP. **(B)** Heatmap displaying the log2 ratios of the DEGs shared in MNP upon FL13 and Lena infection versus control as shown in **Figure 5A**. **(C)** FL13 classical, FL13 ML, and Lena classical analysis compared with IPA. Heatmap displaying selected functional annotations from IPA found to be enriched in one or more DEG lists identified in FL13 or Lena infected versus control MNP, according to different methodologies (conventional vs. ML approaches). Hierarchical clustering was performed with Morpheus (Broad Institute) using the One minus Pearson correlation and the average linkage method.

Only 12 out of the 23 expressed genes found in FL13-infected cells were common with Lena-infected cells (**Figure 5A**), including 8 genes with the same pattern of expression (down-regulated: *RASSFA, SLC46A2, CCDC153, PDZD3*, and up-regulated: *WDR76, BCOR, POGZ, FIGNL1*) and 4 of them showing opposite patterns (*GRHPR* and *CYT B-C1-10, SHARPIN, CYC1*) down-regulated in FL13 and up-regulated in Lena (**Figure 5B**) (**Supplementary Table 4**). Interestingly, *CYC1* and *CYT B-C1-10* are both involved in the mitochondrial respiratory chains, and similarly, *GRHPR* has an oxidoreductase activity. These three genes were all down-regulated during FL13 infection and consistent in pattern to the *in vivo* cell subtypes (PIM, moDC, and cDC1 and 2). Conversely, Lena infection showed up-regulation of these genes. Finally, *NDUFB6*, involved in the mitochondrial respiratory chain complex, was down-regulated during *in vitro* FL13 infection and in most of the *in vivo* infected cells (macrophages and cDC subtypes) but showed the opposite pattern in Lena (data not shown).

Of note, three genes (*ATG5, COX5A, COX5B*) discovered with ML were additionally validated in all the *in vivo* mononuclear phagocyte subsets by qPCR (**Supplementary Table 4**), confirming the accuracy of this data mining method.

### Functional Analyses of FL13 and Lena *In Vitro* Infection

First, an ML approach was used to unravel other potentially hidden intracellular pathways modulated by FL13 infection. In the case of Lena infection, the conventional analysis had a high discovery resolution (4,556 DEGs; **Supplementary Table 3C**), and the ML approach was “overfitting” even upon a data dimensionality reduction (data not shown).

By SMO classifier, decision tree algorithms, and MLP using WEKA software in a data dimensionality reduction, the ML identified 900 relevant genes in the FL13 data set. The best ML classifier performance was reached using around 2,000 genes (Correct classification 100%, Kappa Statistic 1, AUROC 1) and dropped after the removal of the first 900 classified genes (Correct classification range 33.00%–66.66%, Kappa statistic range 0.00–0.40, AUROC range 0.34–0.75). The correct classification of the randomized data sets (negative control of machine learning) ranged from 43.75% to 48.75%, which is around the predicted value after random assignment into two groups (50%). Kappa statistics ranged from -0.03 to 0.00, and AUROC ranged from 0.42 to 0.47.

Importantly, all the 21 genes already obtained by conventional analysis in both the *in vivo* and *in vitro* FL13 data sets were included in the list of genes discovered by ML.

The IPA of the genes obtained by ML for the contrast FL13 MNP versus mock (**Figure 5C** *in vitro* FL ML) was more powerful than the conventional approach (**Figure 5C** *in vitro* FL) with additional pathways, such as GADD45 signaling, TREM1 and EIF2 signaling, and other pathways related to cell cycle regulation and DNA damage response identified (**Figure 5C**). As expected, Lena infection was much more potent in the up-regulation of several inflammatory, interferon, and apoptosis pathways compared with FL13 that lacked such types of responses.

The results on FL13 (obtained by ML) were compared to those obtained on Lena (by conventional approach) by the analysis of pathway enrichment (contrast Lena MNP vs. mock; **Figure 5C**). The two comparisons shared 18 pathways, displaying various enrichment values with only 9 pathways showing significant differences (data not shown). In particular, FL13 infection presented a reduction in the oxidative mitochondrial metabolism (both oxidative phosphorylation and citric acid cycle, **Figure 5C**, **Supplementary Figure 5**), whereas Lena mostly modulated the aerobic glycolysis (AKT/mTOR/PI3K) pathways (**Figure 5C**). The sirtuin pathway, related to an epigenetic-inflammation-suppressive pathway was modulated in both FL13 and Lena (**Figure 5C**).

When compared to the meta-analysis of the PRRSV host response (13), the FL13 analysis by ML highlighted the previously identified oxidative phosphorylation and mitochondrial dysfunction, and the TREM1 signaling was consistent with one of the top five canonical pathways of the pig specific response to PRRSV (13) (**Figure 5C**). The analysis of the Lena data set pointed out mTOR signaling and regulation of eIF4 and p70S6K signaling as additional pathways shared with the global pig immune response and the toll-like receptor signaling with the pig-specific response to PRRSV (13). In addition, a total number of 191 genes were shared by Badaoui’s gene list (13) and Lena versus ctr (exact Fisher’s *F* test *P*-value < 0.05).

Thus, ML performed better than GSEA to expand the limited findings obtained by conventional analysis (**Figure 5**, **Supplementary Figure 5**) as well as to reveal genes and intracellular pathways shared or not between FL13 and Lena and supported by previous studies (13).

## Discussion

AM have been the most studied population during PRRSV infection, but high-resolution expression profiling of lung primary parenchymal subpopulations, recognized as critical components of the innate immune responses against viruses, is missing. Here, we evaluated the transcriptomes of *in vivo* FL13-infected lung MNP subpopulations and *in vitro* FL13- and Lena-infected parenchymal MNP.

### Genetic Signatures of Porcine Lung MNP

As a follow-up to our previous studies (9, 25, 26), we confirmed the different identities of the porcine lung MNP isolated from *in vivo* PRRSV-1 FL13-infected animals with a new sorting strategy. We established cell-specific expression profiles that were subsequently validated with their murine counterparts with the exception of PIM. Indeed, PIM represents a unique population present in the *Laurasiatheria* superorder, including pigs and other livestock mammals but not mice and humans (62). AM and PIM showed similar transcriptomic signatures, highlighting their probable common origin as previously proposed (5). The genetic signatures of PIM, cDC, and moDC at steady state and upon PRRSV-1.1 infection add up to the knowledge of relevant parenchymal MNP subsets for future insights into PRRSV and for comparative studies with other pig respiratory diseases.

### Effect of FL13 *In Vivo* Infection on Different Mononuclear Phagocyte Subsets

In agreement with its low virulence, FL13 had moderate effects on every cell subtype. Both AM and PIM confirmed the highest rate of infection compared to the other MNP subsets, known for being weakly susceptible, or not at all, to *in vivo* PRRSV-1 infection (7, 9). FL13-PIM showed a lower gene modulation (around 3-fold fewer DEGs) compared to FL13-AM, suggesting a different level of activation induced by FL13 despite the similar infection rate (**Supplementary Figure 1B**). As expected, we identified DEGs associated with apoptosis and down-regulation of proliferation in FL13-infected macrophages, especially in AM.

The DC subsets shared an anti-apoptotic profile (**Figure 2**) and the down-regulation of *ATP2C1*, a gene previously shown to correlate with resistance and decreasing viral spread in other viral infections (paramyxovirus, togavirus, flavivirus) (56). cDC2 and moDC also shared the up-regulation of *ABCF3*, an antiviral gene that, when silenced, was correlated with increased replication of flavivirus (59). Despite those interesting findings on the potential DC mechanisms to control PRRSV-1 replication, their susceptibility is probably related to a multifactorial genetic modulation, including the low or null expression of the main PRRSV receptor CD163 and the tuning of apoptosis. Further studies are needed to evaluate the specificity of DC resistance together with *ATP2C1* and *ABCF3* roles. Interestingly, cDC and moDC clustered separately at steady state, confirming our previous observation (52), but displayed similar FL13 responses, especially between moDC and cDC2, pointing out a convergent genetic reprogramming despite their different origin, as previously described in mouse and human (63).

Despite the slow replication kinetics of FL13 likely contributing to the cellular gene expression, the capacity to not trigger a strong immune response and to keep antiviral and inflammatory functions silent could be one of the immune evasion strategies used by PRRSV1.1 strains, such as FL13, to maintain its fitness. However, given the lack of a clear transcriptomic segregation between infected and control cells, the actual impact of these putative mechanisms on the different cell types remains difficult to assess.

### Comparative Analysis of FL13 and Lena Infections

Although FL13 cellular reprogramming was moderate or even silent, Lena *in vitro* infection induced the typical cellular profiles of a virulent strain, i.e., high replication rate, massive gene expression, and activation of the immune responses as previously described in different HP strains (3, 5, 9, 61, 64, 65). Thanks to a more complete analysis performed after ML, we could identify molecular signatures pointing to different modulatory mechanisms by the two strains.

It has been recently shown that NSP1α protein from highly pathogenic PRRSV-2 inhibited NF-kB activation by targeting the linear ubiquitin chain assembly complex (LUBAC) composed by the SHARPIN, HOIP, and HOIL-1L subunit (66). In particular*, SHARPIN* has been associated with NFkB activation and described as essential for cytokine production and induction of Th1 differentiation by DC (67, 68). In our data, *SHARPIN* was up-regulated *in vitro* by Lena infection but was consistently down-regulated by FL13 both *in vivo* (PIM, moDC, and cDC1 and 2) and *in vitro*. Thus, our data are consistent with previous reports, and they may explain the weak Th1 priming in FL13 infection as opposed to the strong Th1 induction profile in Lena infection previously observed in these cells (9). Future studies comparing the behavior of plasmacytoid DC upon FL13 and Lena infections may provide additional insights into antiviral responses.

The cellular redox homeostasis emerges as an important molecular signature of cell impairment upon PRRSV-1 infection, and our *in vitro* findings suggest distinctive pathogenic mechanisms of the two strains in the alteration of the mitochondrial function and in the modulation of the cellular metabolism (**Figure 5**, **Supplementary Table 3**). Mitochondria are the cellular “power plants” and are involved in a range of several intracellular functions, including regulation of redox homeostasis and cell fate (69). Previously, oxidative stress has been described to correlate with immune response impairment by alteration of the macrophage respiratory burst (70) and by modification of the reducing milieu in the immunological synapse between DC and T cells (71).

Oxidative stress appears to be induced by both PRRSV-1 strains through different pathways. Among the several cellular functions and pathways modulated by Lena (**Figure 5C**, **Supplementary Figure 4**), mTOR is a major host cell signaling pathway that regulates protein synthesis, cell growth, proliferation, and survival. It is also established that many viruses exploit this signaling cascade for their own benefit. Previous studies have pointed out the role of mTOR associated with repression of type I IFN during PRRSV (72, 73) and the induction of apoptosis through a mitochondria-mediated pathway (74). Lena showed a clear modulation of mTOR signaling that favors glycolysis, inflammation, cytokine production, and the capacity to prime T cells toward Th1 (75) and no involvement in the oxidative phosphorylation pathway. In contrast, our findings point to a decreased oxidative phosphorylation and citric acid cycle induced by FL13 through a different pattern of gene suppression. However, it remains difficult to untangle the respective roles of the low replication rate of FL13 and its active down-modulation of the immune response in the resulting immune evasion capacity of the virus.

Viruses can induce different metabolic reprogramming that depends on the host cell type that is infected (76). Many cellular disturbances, such as redox imbalance, cause accumulation of misfolded proteins or unfolded proteins, which, in turn, leads to activation of an evolutionary conserved signaling pathway called the unfolded protein response (UPR). Severe or prolonged activation of the UPR can cause cell death induction that is involved in the pathogenesis of various diseases (77). The induction of UPR has been found not only contributing to PRRSV-induced apoptosis in host cells (55, 78), but also involving in the regulation of virus replication and dysregulation of AM cytokine production (79). In our data, infection of lung parenchymal MNP involved the UPR pathway and apoptosis, and the profile was more significant during Lena than FL13 infection.

## Conclusion

This study shows, for the first time, the different transcriptomic profiles identified in lung MNP upon *in vivo* infection with a PRRSV-1 strain. The distinctive molecular signatures and pathways of cellular reprogramming found *in vitro* are suggestive of a different mechanism of pathogenesis driven by low and highly pathogenic strains. As such, this work paves the way for future mechanistic studies to evaluate the role of virus genetics and restriction factors in driving such distinctive cellular expression beneath the pathogenesis of the infection.

## Data Availability Statement

The datasets presented in this study can be found in online repositories. The names of the repository/repositories and accession number(s) can be found in the article/**Supplementary Material**.

## Ethics Statement

The animal study was reviewed and approved by the French Ministry for Research (authorization no. 2015051418327338) and protocols were approved by the national ethics committee (APAFIS#413).

## Author contributions

EC performed most of the experiments, conceptualization, machine learning, data analysis, and wrote the manuscript. ElB, EdB, and NB performed the *in vitro* experiments. JP, AP, and OB supported *in vivo* experiments. ElB and EdB supported the samples collection and processing. AM and LJ performed the library preparation, sequencing and primary RNAseq data analysis. MM performed the design of qPCR primers, PCA and functional analysis of the data. CB performed the RT-qPCR for validations. MB supported the cell-sorting. CU performed the RT-qPCR for the viral loads in swabs and sera. TPVM performed the interspecies transcriptomic comparison study and curated data visualization. EG, IS, and NB provided financial supports. NB and EG conceived the study, supervised the working program, and contributed to writing the manuscript. All authors contributed to the article and approved the submitted version.

## Funding

This work was funded by the Animal Health and Welfare ERA-Net (anihwa)-project KILLeuPRRSV.

The present work has benefited from the core facilities of Imagerie-Gif, (http://www.i2bc.paris-saclay.fr), member of IBiSA (http://www.ibisa.net). It was supported by the Programme d’Investissement d’Avenir France-BioImaging (Agence Nationale de la Recherche ANR-10-ISBN-04-01), and the Labex Saclay Plant Science (Agence Nationale de la recherche, ANR-11-IDEX-0003-02).

EC was supported by the Animal Health and Welfare ERANet (anihwa)-project KILLeuPRRSV and the AgreenSkills+ fellowship program, which has received funding from the EU’s Seventh Framework Programme under grant agreement N◦ FP7-609398 (AgreenSkills+ contract). ElB was supported by H2020 SAPHIR. The École normale supérieure genomic core facility was supported by the France Génomique national infrastructure, funded as part of the “Investissements d’Avenir” program managed by the Agence Nationale de la Recherche (contract ANR-10-INBS-09).

## Conflict of Interest

The authors declare that the research was conducted in the absence of any commercial or financial relationships that could be construed as a potential conflict of interest.
