# Supplementary material for: Distinctive Cellular and Metabolic Reprogramming in Porcine Lung Mononuclear Phagocytes Infected With Type 1 PRRSV Strains
Source: Front Immunol. 2020 Dec 7;11:588411. doi: 10.3389/fimmu.2020.588411 (PMC7750501; doi:10.3389/fimmu.2020.588411)
Supplement: Supplementary file 1 [file DataSheet_1.zip › Data Sheet 1.PDF]

Supplementary Figure 1.

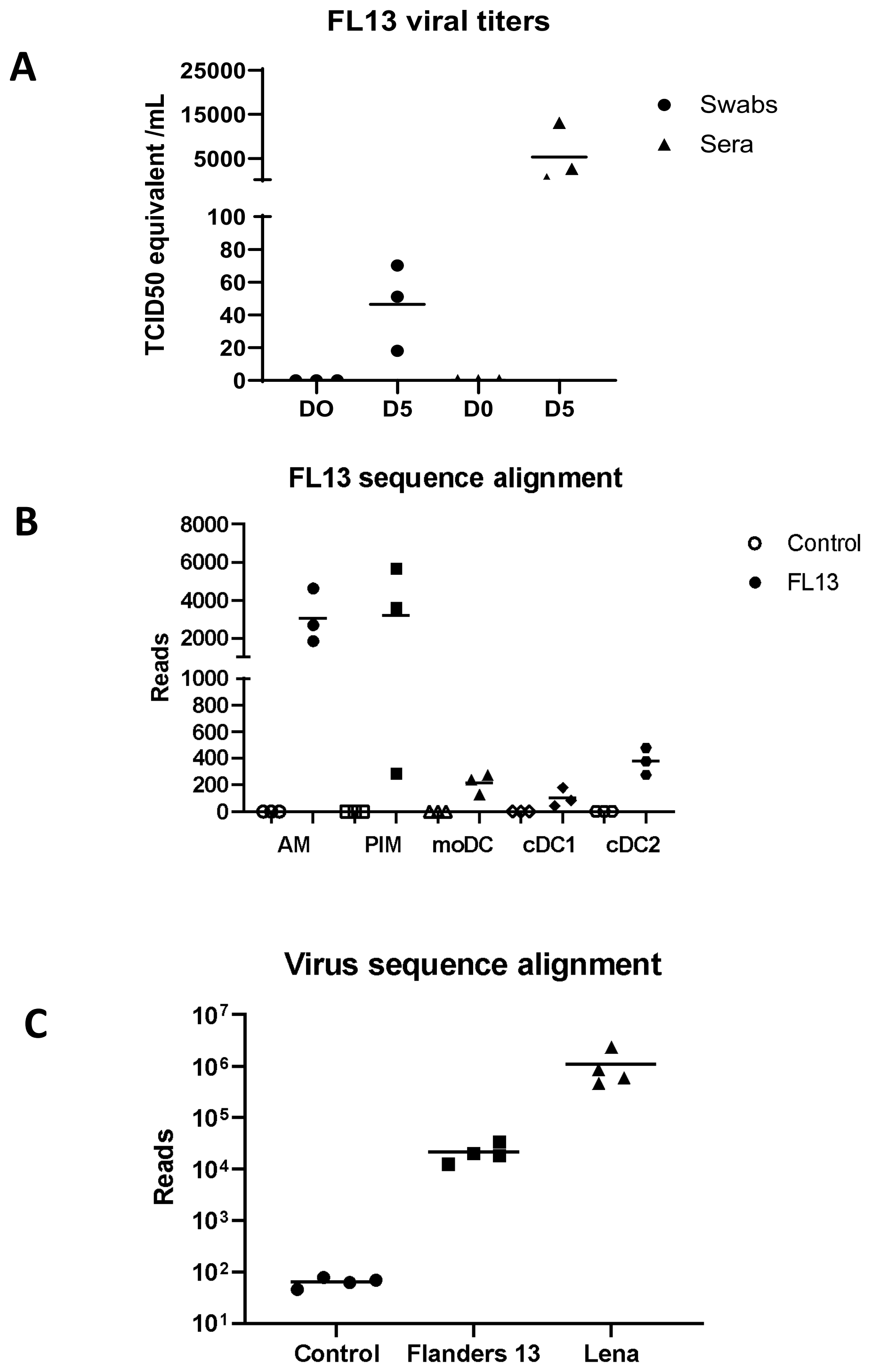

Sup. Fig. 1. FL13 *in vivo* and *in vitro* infection. A-B) Animals were infected with FL13 5x105 TCID50/animl for 5 days. A) Detection of viral transcripts in swabs and sera of FL13-infected and control animals was assessed by RT-qPCR at day 0 and day 5 post infection. Primers designed on viral nucleocapsid protein (N) were used to detect viral transcripts. B) Reads aligned to FL13 genome sequence present in lung MNP subpopulations sorted from FL13 infected and control animals at day 5 post infection. N=3 C) FL13 and Lena *in vitro* infection. Enriched lung MNP were *in vitro* infected at MOI= 0.5 with FL13 and LENA PRRSV strains. After 24h infection, the cell pellet were collected and RNAseq performed. Reads aligned to FL13 and Lena genome sequences present in parenchymal enriched MNP. N=4

Supplementary Figure 2

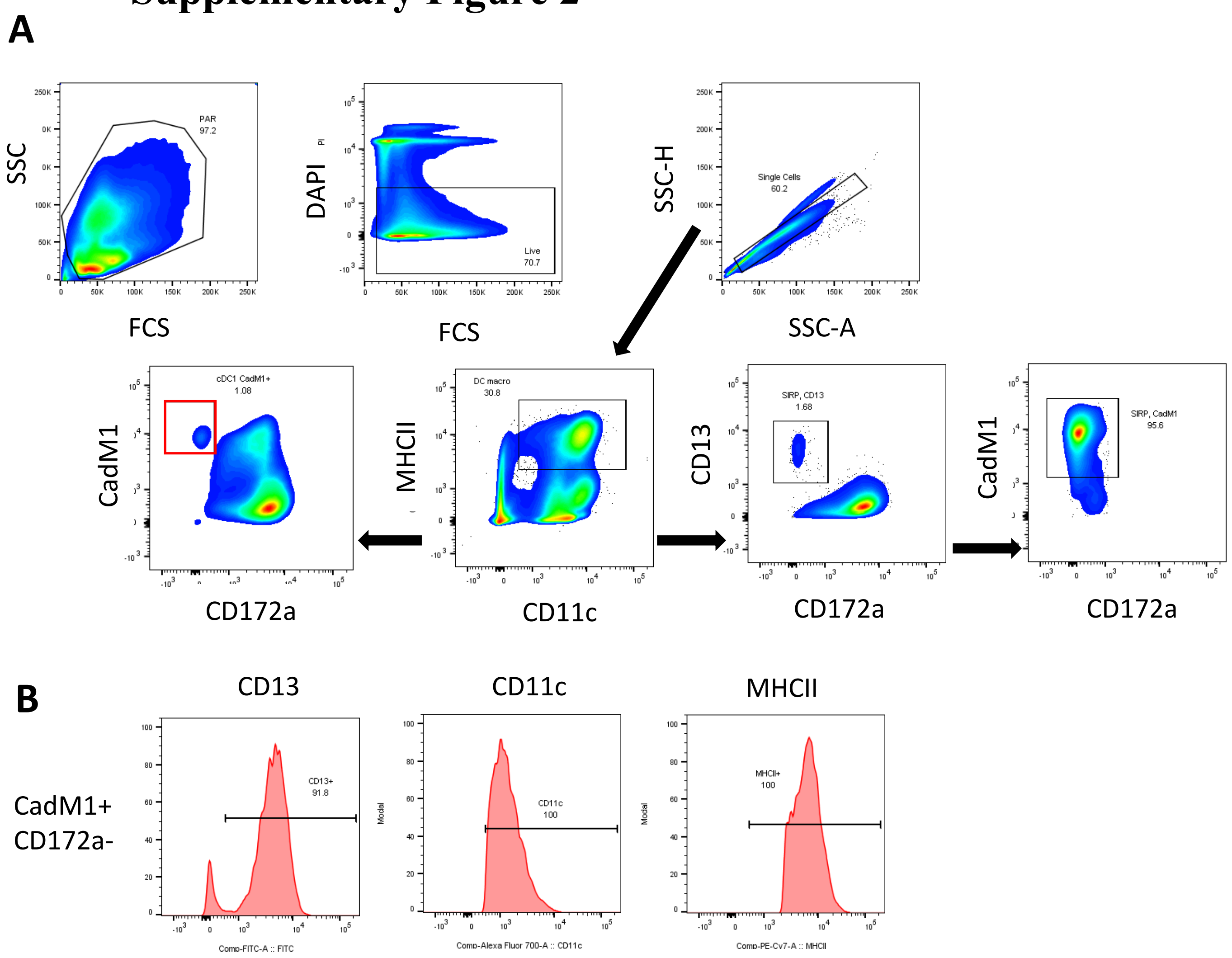

Sup. Fig. 2. CadM1+ cDC1 cells are positive for CD13 marker. A) cDC1 were defined by using MHC-II/CD11c/CD172a/CadM1 or CD13 markers. Each graph is the offspring of the previous gate from left to right or following the arrows. (B) CD13, CD11c and MHCII expression in CadM1+ CD172- cells (red box). CadM1+ CD172- cDC1 are CD13 high, CD11c high, MHCII high.

Supplementary Figure 3.

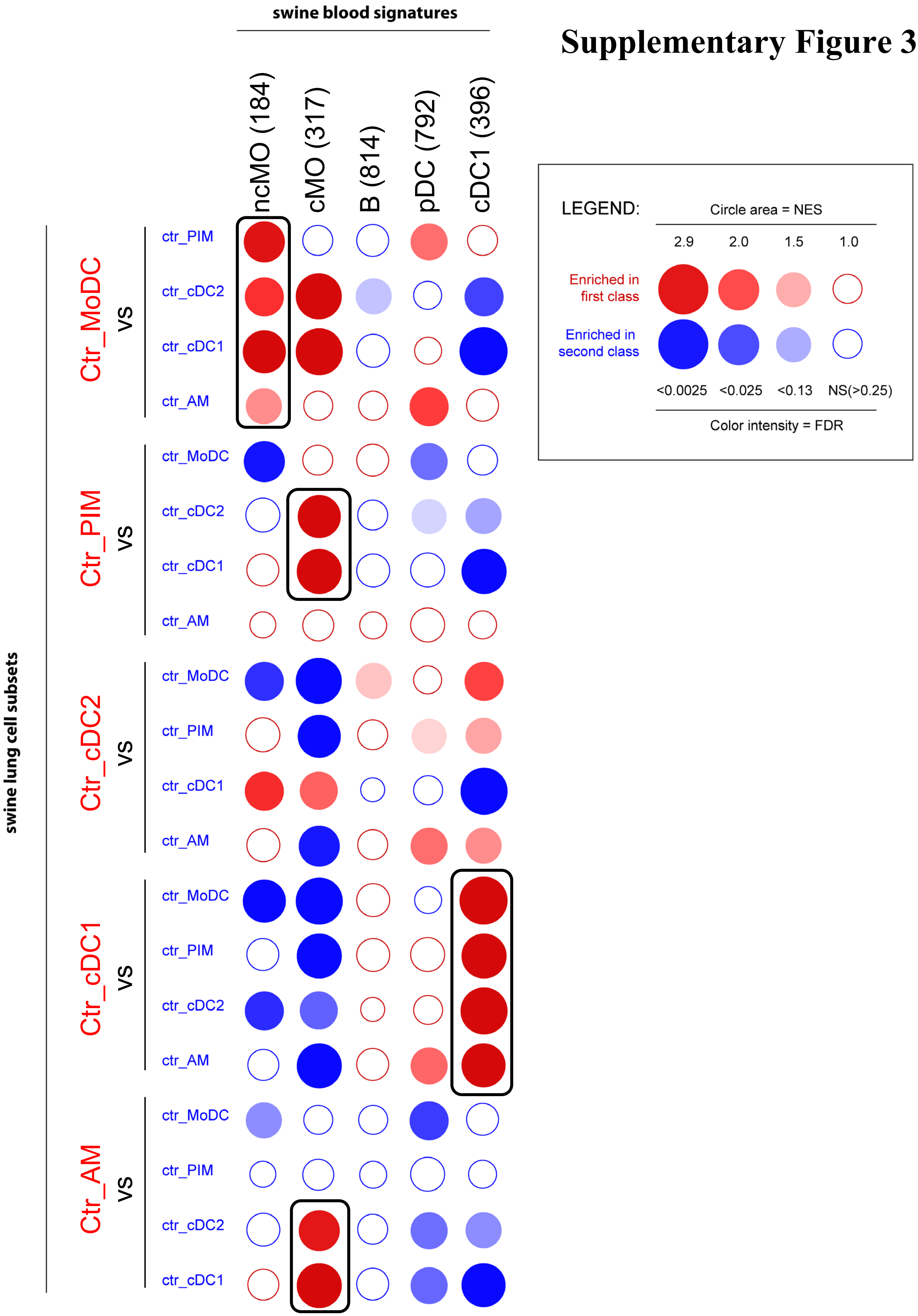

Sup. Fig. 3. Analysis of the homologies between swine lung MNP subsets and swine blood subsets (Vu Manh et al 2015) by high throughput GSEA using BubbleGUM. Transcriptomic signatures generated from swine blood subsets using GeneSign (MinTest/MaxRef>1x) were assessed for enrichment in all possible pairwise comparisons between the control swine lung cell populations using BubbleMap. Data are represented as bubbles, bigger and darker for stronger and more significant enrichment, in a color matching that of the population in which the signature was enriched. Boxes correspond to enrichments of interest. ncMO: non-classical Monocytes; cMO: classical Monocytes.

Supplementary Figure 4.

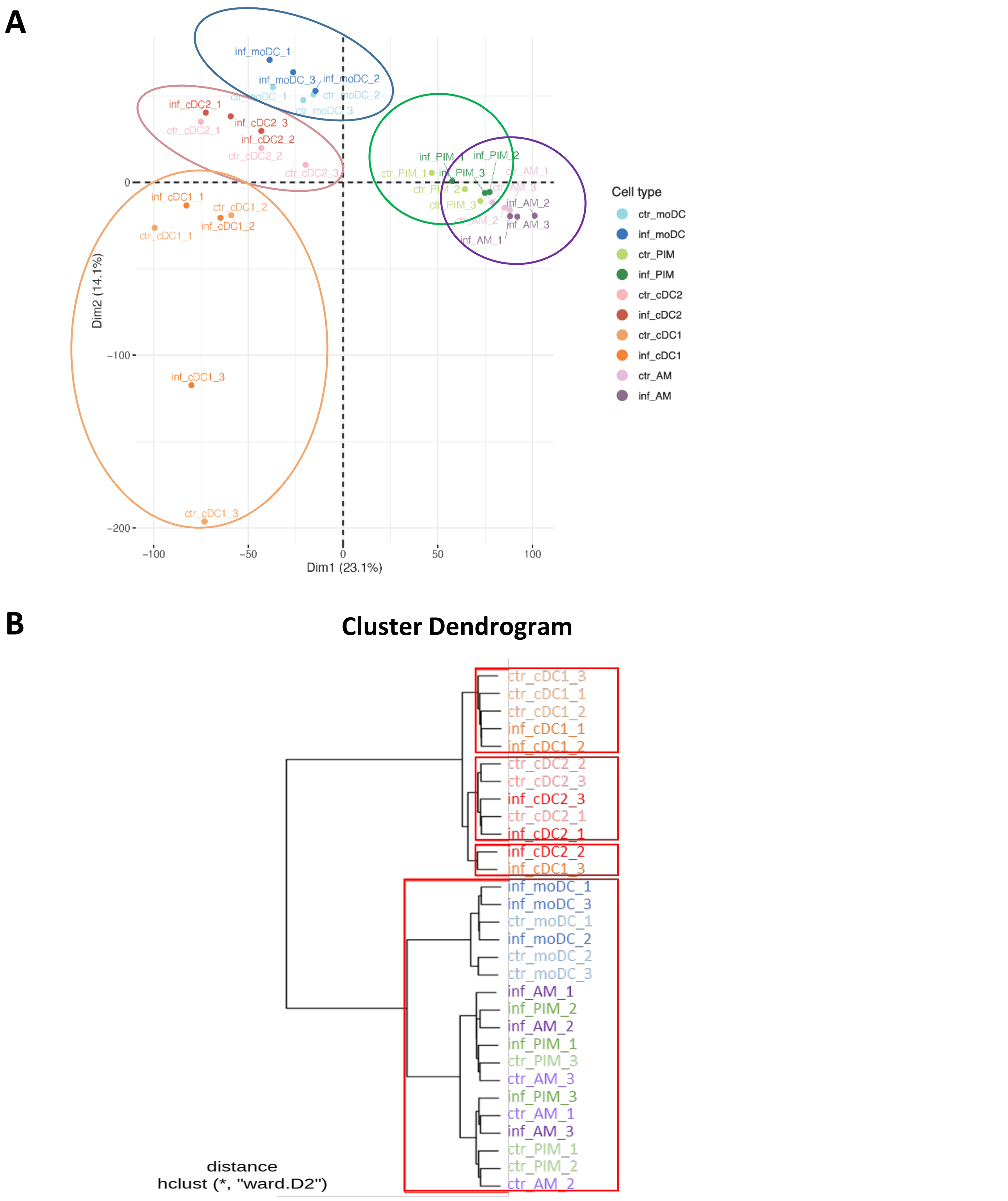

Sup. Fig. 4. FL13 *in vivo* infection. Principal component analysis (PCA) and hierarchical cluster (h-cluster) of FL13 infected and control MNP subsets. A) Plot of the first two components of the PCA analysis based on the data obtained from the sorted cell types. B) Unsupervised hierarchical clustering on the transcriptome of sorted MNP from control and infected animals was performed as described in Figure 1C. Clusters with AU > = 85% are indicated by the rectangles.

Supplementary Figure 5

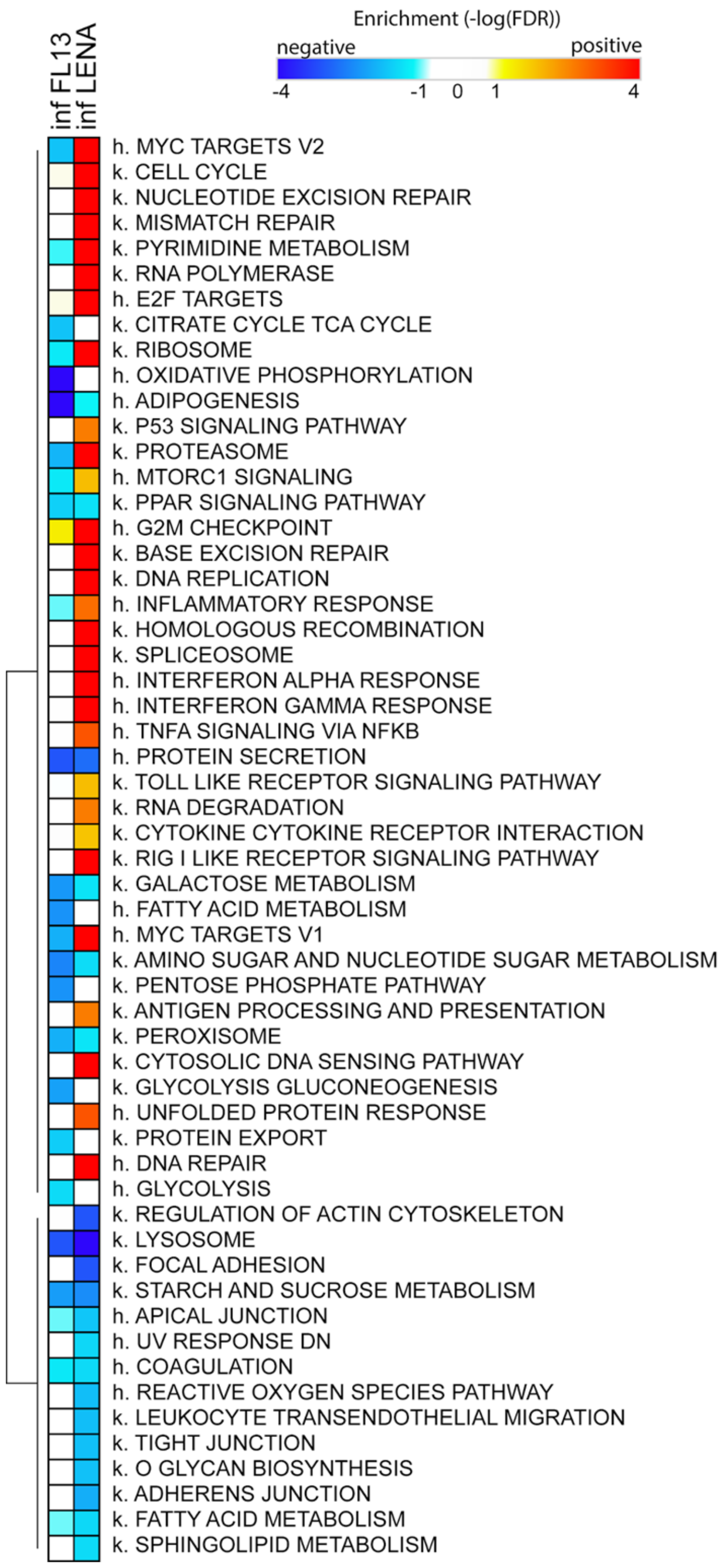

Sup. Fig. 5. **Enrichment of functional annotations in MNP upon FL13 or Lena in vitro infection, using GSEA.** Heatmap displaying selected genesets from MSigDB found to be enriched by GSEA upon FL13 or Lena *in vitro* infection. Genesets found up- and down-regulated upon infection have positive (red) and negative (blue) enrichments respectively. Genesets preceded with k. come from the Kegg pathway database, and genesets preceded with h. come from the Hallmark collection of the MSigDB.
Hierarchical clustering was performed with Morpheus (Broad Institute) using the One minus Pearson correlation and the average linkage method.
