# Supplementary material for: Distinctive Cellular and Metabolic Reprogramming in Porcine Lung Mononuclear Phagocytes Infected With Type 1 PRRSV Strains
Source: Front Immunol. 2020 Dec 7;11:588411. doi: 10.3389/fimmu.2020.588411 (PMC7750501; doi:10.3389/fimmu.2020.588411)
Supplement: Supplementary file 1 [file DataSheet_1.zip › Data Sheet 2.PDF]

Supplementary Table 1.

Antibody	Clone	Isotype	Specie	Working concentration	Supplier
**CD1-FITC**	76-7-4	IgG2a	Mouse	4µg/ml	Southern Biotech
**CD11c biot**	3A8	IgG1	Mouse	2µg/ml	Homemade
**CD13**	T35	IgG1	Mouse	2.5 µg/ml	Homemade
**CD163-PE**	2A10/11	IgG1	Mouse	5µg/ml	Biorad
**CD172a/Sirpα**	74-22-15A	IgG2b	Mouse	4µg/ml	WSU
**MHCII (SLAII)**	MSA3	IgG2a	Mouse	4µg/ml	WSU

Supplementary Table 1. Antibodies used for cell sorting.

Supplementary Table 2.

Gene ID	Sense	Sequence (5'-3')	Lenght (pb)
**GRHPR**	FOR	GGATGAAGAAAACGGCTGTGTT	22
**GRHPR**	REV	CCAGTCCAGCAGCTGCAAT	19
**ENS13436 (CYT B-C1-10)**	FOR	CGCGAGCTGGCCAGAA	16
**ENS13436 (CYT B-C1-10)**	REV	TGATGTAGGGCACCCAATCC	20
**SHARPIN**	FOR	TGCATCCTCTGCCCATGTC	19
**SHARPIN**	REV	AGGGAAGCCAAACTCTGAGAAGA	23
**CYC1**	FOR	CGCGGCCTCCTCTCATC	17
**CYC1**	REV	CACACCTGCTTGTATACCTGGAAA	24
**ATG5**	FOR	TGGATGGGTTTACAAAATGACAGAT	25
**ATG5**	REV	GAAATCCATTTTCTTCTGCAGGAT	24
**COX5A**	FOR	TGAACACGCTTGTTGGCTATG	21
**COX5A**	REV	TCCTGCTTTGTCCTTAACAACCT	23
**COX5B**	FOR	CTGGAGAGGGAGGTCATGATG	21
**COX5B**	REV	GGTACCTGAGGCTGCCTTTG	20
**ATP2C1**	FOR	GGTGCTTATGAACAGGTGATTAAGTAT T	28
**ATP2C1**	REV	AGAAGCCAAAGCAAGAACTCTGA	23
**DTX4**	FOR	TGCCTGGTTGCCATGTACAA	20
**DTX4**	REV	CCATAAATGGTCTTACAGGTTGGA	24
**BIRC5**	FOR	CCGCATCTCCACATTTAAGAACT	23
**BIRC5**	REV	TCAGTGGGACAGTGGATGAAAC	22
**STMN1**	FOR	AGAAAGACGCAAGTCCCATGA	21
**STMN1**	REV	TGCTCCCGTTTCTCAGCAA	19
**GAPDH**	FOR	CAACGACCACTTCGTCAAGCT	21
**GAPDH**	REV	CACCCTGTTGCTGTAGCCAAA	21

Supplementary Table 2. Primers used for transcriptomic validations

Supplementary Table 3.

A

DEGs between control cell type	DE genes - total	DE genes - with human gene symbol
ctr-cDC2 vs ctr-cDC1	2950	2392
ctr-moDC vs ctr-cDC1	4478	3824
ctr-cDC1 vs ctr-AM	7863	6682
ctr-cDC1 vs ctr-PIM	6119	5221
ctr-moDC vs ctr-cDC2	2174	1908
ctr-cDC2 vs ctr-AM	6661	5717
ctr-cDC2 vs ctr-PIM	4817	4193
ctr moDC vs ctr AM	6337	5487
ctr moDC vs ctr PIM	4040	3506
ctr-PIM vs ctr-AM	658	557

B

DEGs across contrasts (infected vs control)	DE genes - total	DE genes - with human gene symbol
inf-AM vs ctr-AM	101	86
inf-PIM vs ctr-PIM	35	33
inf-cDC1 vs ctr-cDC1	157	135
inf-cDC2 vs ctr-cDC2	78	69
inf-moDC vs ctr-moDC	106	96

C

DEGs between treatments	DE genes - total	DE genes - with human gene symbol
In vitro FL vs Ctr	24	21
In vitro FL vs Ctr ML	900	739
In vitro Lena vs Ctr	4556	3958
In vitro FL vs Lena	4048	3547

Supplementary Table 3. Summary of the main differential analysis contrasts, also including the results obtained with Machine Learning (indicated as 'ML' in the table). For each contrast, the total number of genes obtained is given in the first column, while the second column lists the genes for which the human ortholog was found. A) Summary of the main contrasts obtained from the in vivo experiment between cell subtypes. (B) Summary of the main contrasts obtained from the in vivo experiment between infected and control cells. C) Summary of the main contrasts obtained from the in vitro experiment.

Supplementary Table 4.

Type of sample	Gene and cell type	qPCR data		RNAseq data	
		FC	SE	FC	SE
In vivo	ATG5 moDC	0.96	0.16	1.28	0.03
	ATG5 PIM	0.99	0.11	0.95	0.03
	ATG5 cDC2	2.1	0.64	0.93	0.03
	ATG5 cDC1	0.86	0.04	0.92	0.03
	ATG5 AM	0.59	0.11	0.87	0.03
	ATP2C1 moDC	0.13	0.05	0.23	0.24
	ATP2C1 PIM	0.57	0.41	0.4	0.24
	ATP2C1 cDC2	0.72	0.47	0.29	0.24
	ATP2C1 AM	0.2	0.12	0.31	0.24
	BIRC5 moDC	0.5	0.16	0.68	0.17
	BIRC5 PIM	0.3	0.03	0.19	0.17
	BIRC5 cDC2	0.3	0.68	0.81	0.17
	BIRC5 AM	0.18	0.12	0.31	0.17
	COX5A moDC	0.8	0.03	1.01	0.04
	COX5A PIM	0.98	0.1	1.00	0.04
	COX5A cDC2	2.4	1.39	0.92	0.04
	COX5A cDC1	1.01	0.18	0.9	0.04
	COX5A AM	0.48	0.06	0.93	0.04
	COX5B moDC	0.96	0.12	1.28	0.06
	COX5B PIM	1.08	0.09	1.17	0.06
	COX5B cDC2	1.96	0.66	0.98	0.06
	COX5B cDC1	1.18	0.09	0.99	0.06
	COX5B AM	0.75	0.04	0.96	0.06
	DTX4 moDC	0.38	0.09	0.49	0.07
	DTX4 PIM	0.88	0.18	0.97	0.07
	DTX4 cDC2	0.32	0.07	0.47	0.07
	DTX4 cDC1	1.81	0.01	0.81	0.07
	DTX4 AM	0.48	0.05	0.91	0.07
	GRHPR moDC	0.69	0.1	1.2	0.12
	GRHPR PIM	0.83	0.23	0.84	0.12
	GRHPR cDC2	1.41	1.01	1.32	0.12
	GRHPR cDC1	0.68	0.02	0.54	0.12
	GRHPR AM	0.51	0.1	0.87	0.12
	STMN1 moDC	0.97	0.18	1.16	0.12
	STMN1 PIM	0.33	0.07	0.28	0.12
	STMN1 cDC2	1.76	1.03	1.05	0.12
	STMN1 cDC1	1.19	0.04	1.13	0.12
	STMN1 AM	0.19	0.05	0.28	0.12
In vitro	CYC1 Flanders	1.14	0.32	0.77	0.01
	CYC1 Lena	1.04	0.14	1.15	0.01
	ENS13436 Flanders	1.16	0.08	0.77	0.01
	ENS13436 Lena	1.27	0.06	1.38	0.01
	SHARPIN Flanders	1.27	0.4	0.78	0.01
	SHARPIN Lena	1.05	0.19	1.2	0.01

Supplementary Table 4. Validation of the RNAseq data by qPCR. Expression values are indicated as fold changes (FC) and standard error (SE) between infected samples and controls. The correlation between RNAseq and qPCR data calculated using the Spearman's Rho test was rs = 0.75501 at p-value (2-tailed) of 0.00.
